# Empowering Futures: Intersecting Comprehensive Sexual Education for Children and Adolescents With Sustainable Development Goals

**DOI:** 10.7759/cureus.65078

**Published:** 2024-07-22

**Authors:** Nor Faiza Mohammed Tohit, Mainul Haque

**Affiliations:** 1 Department of Community Health, National Defence University of Malaysia, Kuala Lumpur, MYS; 2 Department of Research, Karnavati Scientific Research Center, Karnavati School of Dentistry, Karnavati University, Gandhinagar, IND; 3 Department of Pharmacology and Therapeutics, National Defence University of Malaysia, Kuala Lumpur, MYS

**Keywords:** relationships and consent, emotional health and sexual rights, children and adolescents, environmental sustainability, economic growth, educational impact, health outcomes, gender equality, sustainable development, comprehensive sexual education

## Abstract

This scoping review synthesizes the intersection of comprehensive sexual education (CSE) for children and adolescents with sustainable development goals (SDGs) to assess the potential for empowering future generations. Considering the global challenges in addressing sexual health, this review explores the potential role of CSE in contributing to the achievement of the SDGs, particularly in the context of empowering youth and ensuring their well-being. The review begins by providing a comprehensive overview of CSE, emphasizing its role in promoting informed decision-making, gender equality, and preventing sexual and reproductive health issues among young individuals. It then delves into the overarching framework of the SDGs, mainly focusing on goals related to health, education, gender equality, and sustainable development.

The synthesis examines the alignment and potential synergies between CSE and various SDGs, highlighting how CSE can contribute to outcomes such as improved health and well-being, quality education, gender equality, and reduced inequalities. Furthermore, the review brings attention to potential challenges and barriers in implementing CSE programs in different cultural and socio-economic contexts. Ultimately, this scoping review provides a critical analysis of the intersection between CSE and the SDGs, offering insights into how the comprehensive education of children and adolescents in sexual and reproductive health can play a significant role in advancing sustainable development and empowering future generations worldwide.

## Introduction and background

Comprehensive sexual education (CSE) for children and adolescents is a holistic, evidence-based educational approach that provides young people with the knowledge, skills, attitudes, and values they need to make informed decisions about their sexual and reproductive health and well-being [1,2]. CSE covers various topics, including human development, anatomy and physiology, puberty, reproduction, contraception, sexual orientation, gender identity, relationships, consent, emotional health, and sexual rights [2-4]. According to the latest technical guidelines released by UNESCO in 2018, the following definition is provided: "CSE is a curriculum-based process [that] aims to equip children and young people with…develop respectful social and sexual relationships; consider how their choices affect their well-being and that of others; and understand and ensure the protection of their rights throughout their lives" [5].

The objective of CSE is not only to impart information but also to foster critical thinking, self-awareness, and empathy. It aims to empower young individuals by helping them understand their bodies, relationships, and feelings and by providing them with tools to navigate societal pressures, gender stereotypes, and the complexities of sexual health respectfully and responsibly [6]. CSE promotes the development of skills such as communication, negotiation, and decision-making, which are vital for forming healthy and respectful relationships [6-8].

CSE must be appropriate and culturally relevant, adapting its content to suit its learners' developmental stages and cultural contexts [9]. It engages parents, communities, and schools to create a supportive environment for young people. By equipping children and adolescents with comprehensive and accurate sexual education, CSE is crucial in promoting health, well-being, and gender equality, laying the foundation for informed and responsible adulthood [10].

**Figure 1:**
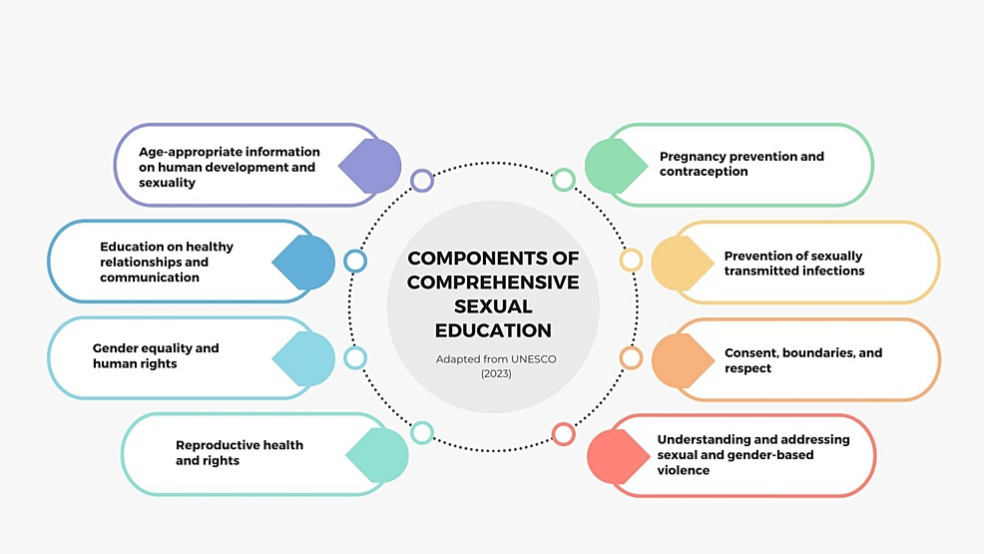
Components of CSE. CSE, comprehensive sexual education Image credit: Nor Faiza Mohammed Tohit

Research consistently demonstrates that CSE can delay the onset of sexual activity, reduce the number of sexual partners, increase the use of contraception, and decrease the incidence of sexually transmitted infections (STIs) and unintended pregnancies [11-15]. In an era of interconnected global challenges and aspirations, the sustainable development goals (SDGs) are a "beacon of hope" and headway and betterment for humanity [16]. Among these goals, ensuring healthy lives and promoting well-being for all at all ages (Goal 3), achieving gender equality, and empowering all women and girls (Goal 5) are central to the agenda of global development [16,17]. A crucial yet often overlooked aspect of these objectives is CSE for children and adolescents [18]. The components of CSE are depicted in Figure 1.

The objectives of this review are multifaceted, aiming to highlight the integral connection between CSE and broader SDGs [19]. First, it seeks to emphasize how CSE promotes good health and well-being by improving sexual and reproductive health outcomes, such as reducing STIs and unintended pregnancies [20]. Additionally, the review underscores the role of CSE in advancing gender equality by challenging harmful gender norms and reducing gender-based violence. It demonstrates how integrating CSE into quality education ensures inclusive and equitable learning opportunities. By empowering individuals, particularly girls, CSE supports poverty alleviation and economic growth by enabling them to pursue educational and vocational opportunities [21].

**Figure 2:**
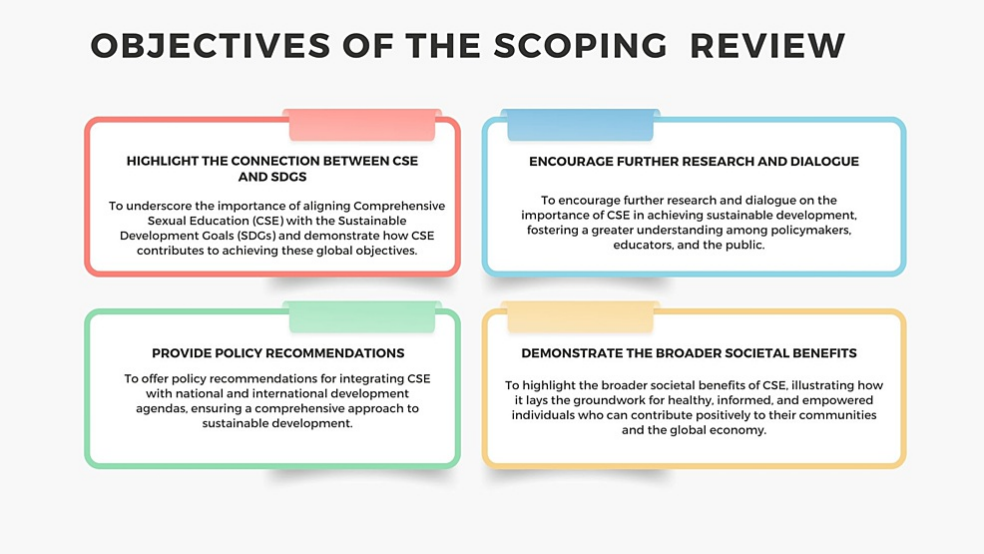
Objectives of the scoping review. Image credit: Nor Faiza Mohammed Tohit

The review advocates for multi-stakeholder partnerships to enhance the effective implementation of CSE programs and highlights CSE's indirect benefits in promoting environmental sustainability [22]. Ultimately, the review offers policy recommendations and encourages further research to foster a comprehensive approach to sustainable development [23]. Figure 2 depicts the objectives of this review.

## Review

Materials and methods

**Figure 3:**
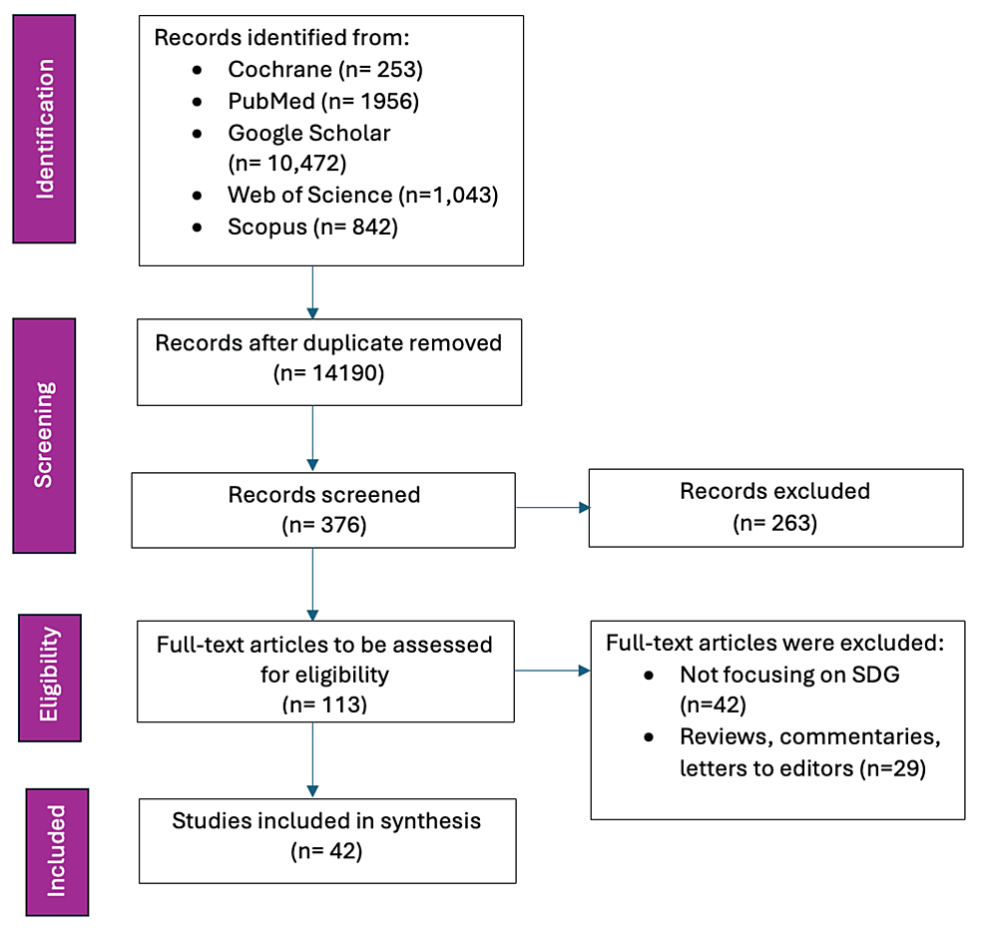
The PRISMA-ScR flowchart on the process of conducting the scoping review. PRISMA-ScR, Preferred Reporting Items for Systematic Reviews and Meta-Analyses Extension for Scoping Reviews Image credit: Nor Faiza Mohammed Tohit

The methodological approach for this scoping review was adapted from standard research practices as described by Arksey and O'Malley (2000) [24]. The framework encompasses identifying the research question; searching for relevant studies; selecting the studies; charting the data; and collating, summarizing, and reporting the results. The process of conducting the review is shown in Figure 3. The primary research question guiding this review was to investigate the current scope of literature on the alignment between CSE and SDGs.

A systematic search was conducted across multiple academic databases, including PubMed, Google Scholar, Web of Science, and Scopus, using a combination of keywords such as "Comprehensive Sexual Education," AND "sustainable development," AND "CSE impact," AND "health outcomes," AND "gender equality," AND "education," AND "economic development," AND "environmental sustainability" with Boolean operators to refine the search. The inclusion and exclusion criteria are depicted in Table 1.

**Table 1:** Details regarding the selection of papers' inclusion and exclusion criteria. CSE, comprehensive sexual education

Inclusion Criteria	Exclusion Criteria
Published in English	Papers: opinion pieces and editorials without substantial data.
Published within the last 10 years (2014-2024)	Empirical research, abstracts only, conference posters.
Research that explicitly connects CSE with sustainable development aspects such as health, education, gender equality, economic growth, environmental sustainability, peer-reviewed articles, reports of governmental and non-governmental organizations, and policy analysis papers.	Other formats lack sufficient detail for complete analysis.
-	Duplicates of previously identified studies were excluded unless they provided unique data or insights.

Titles and abstracts of studies were initially reviewed, followed by full-text screening to ensure relevance based on the inclusion and exclusion criteria. A data extraction form was developed to systematically chart relevant information from the selected studies, including author(s), year of publication, study location, objectives, methodology, and key findings related to CSE and sustainable development. Data extraction was conducted by one reviewer and verified by another to ensure accuracy. Extracted data were categorized based on significant themes: health impact, gender equality, educational outcomes, economic impact, and environmental sustainability.

Critical materials include over 100 peer-reviewed articles and reports from reputable sources such as the World Health Organization (WHO), the United Nations Educational, Scientific and Cultural Organization (UNESCO), and the United Nations Population Fund (UNFPA). Digital tools such as online databases and digital libraries were used for literature search and retrieval, and data were methodically organized to identify key themes and trends.

This review considered biases such as publication bias, language bias, and selection bias [25]. To overcome these biases, comprehensive search strategies using multiple databases minimized publication and language biases. Transparent selection criteria and a systematic approach to study selection, accompanied by independent reviewers, help address selection bias. Additionally, clearly defining the research questions and objectives mitigated potential bias during the review process. By rigorously reviewing and synthesizing existing literature, this scoping review aims to provide a comprehensive understanding of CSE's role in sustainable development and identify directions for future research.

Review of literature

Implementation of CSE Globally

Implementing CSE globally has garnered increasing attention due to its vital role in promoting sexual health, gender equality, and overall well-being [26]. As countries strive to address diverse societal and developmental challenges, the effective global implementation of CSE programs is pivotal for cultivating informed, empowered, and healthier communities [27].

The Netherlands is often cited as a model for CSE implementation. Dutch sexual education starts as early as age four, encompassing age-appropriate topics that evolve as children grow older. Programs emphasize respect, healthy relationships, and informed choices, contributing to the Netherlands seeing some of the lowest rates of teenage pregnancies and sexually transmitted infections (STIs) globally [28].

Sweden has integrated CSE into its national curriculum for over 70 years. In Swedish schools, sexual education covers a wide range of topics, including human rights, gender equality, sexuality, and relationships, ensuring that students gain a holistic understanding of sexual health and well-being. This education approach has fostered a society that values gender equality and sexual health [29].

In Kenya, the National AIDS Control Council, in collaboration with UNESCO and other partners, has developed and implemented the National School Health Policy, which includes CSE components. This initiative aims to equip young people with the knowledge and skills necessary to make informed decisions about their sexual health and to reduce the prevalence of HIV/AIDS and other STIs among adolescents [30].

In Malaysia, CSE has been approached with a culturally sensitive lens, reflecting the country's diverse social and religious backdrop. The Ministry of Education introduced the Reproductive Health and Social Education (PEERS) program in schools as part of the formal curriculum. The program, integrated into subjects like Physical Education and Moral Education starting from primary school, aims to provide students with accurate information about puberty, reproductive health, and the importance of developing respectful relationships. The Malaysian government collaborates with various stakeholders, including non-governmental organizations (NGOs), to enhance the delivery and effectiveness of CSE. For example, the Federation of Reproductive Health Associations, Malaysia (FRHAM) extensively provides supplementary educational materials and training for educators to ensure that the content is age-appropriate and culturally respectful. The focus is on promoting healthy adolescent development and preventing teenage pregnancies and STIs [31].

Despite ongoing efforts, challenges remain, such as cultural taboos around discussing sexual health openly and the need for more comprehensive coverage that includes consent and gender equality [32]. However, the continuing integration of CSE into the education system reflects a growing recognition of its importance in fostering informed and responsible individuals, which aligns with broader health and developmental goals.

In Saudi Arabia, the approach to CSE is considerably conservative, reflecting the country's cultural and religious sensitivities. Traditionally, sex education has not been a formal part of the school curriculum, and discussions around sexual and reproductive health are often limited due to cultural taboos and religious restrictions [32]. However, there have been gradual and cautious steps toward enhancing awareness about sexual health, primarily driven by concerns regarding public health issues such as HIV, STIs, and reproductive health [33]. These efforts are usually embedded within broader health education programs rather than standalone CSE initiatives. For example, the Saudi government has made efforts to include basic information about puberty and reproductive health in the school curriculum, presented in a manner that aligns with Islamic teachings and cultural values.

NGOs and international bodies, such as the UNFPA, have also been subtly influential in pushing toward better sexual health education by providing resources and training to healthcare professionals. They emphasize the importance of educating young people about their bodies and health in a manner that is respectful of cultural norms. Despite these efforts, significant barriers remain, including societal resistance and strict regulations on public discussions about sexuality. As a result, much of the information young people receive about sexual health comes from informal sources, which can sometimes be inaccurate or incomplete. Therefore, while the movement toward CSE in Saudi Arabia is cautious and slow, there is a growing recognition of its importance for the well-being of young individuals within the framework of cultural and religious acceptability [34,35].

South Africa's CSE focuses on addressing the country's high rates of HIV/AIDS and teen pregnancies. The curriculum includes comprehensive information on sexual health, contraception, and HIV prevention. South Africa's CSE Africa also aimed to address gender-based violence and societal norms while promoting reproductive rights and access to healthcare services [36].

Australia's CSE programs emphasize respect, consent, and bodily autonomy. The curriculum addresses a range of topics, including cyber safety, pornography, and healthy relationships, adapting to the evolving needs of young people in a digital age [37]. CSE in Australia aims to equip students with the knowledge and skills to make informed decisions about their sexual and reproductive health, emphasizing inclusivity and diversity and ensuring that the curriculum is sensitive to the needs of all students regardless of their gender or sexual orientation [35,38]. This approach aligns with Australia's broader goal of providing holistic and practical sex education to its youth. These examples illustrate how CSE can be implemented globally, tailored to fit different cultural and societal contexts while achieving common goals of promoting health, well-being, and equality.

The Integral Role of CSE in Achieving SDG​​​​​​s

The SDGs are an ambitious set of 17 interconnected global objectives established by the United Nations in September 2015 as part of the 2030 Agenda for Sustainable Development [16,39]. These goals were formulated to address a vast range of urgent global challenges, encapsulating economic, social, and environmental dimensions of sustainable development, thereby paving the path for a more peaceful, prosperous, and equitable world [40]. The SDGs build on the success and lessons learned from the millennium development goals (MDGs), shifting toward a more comprehensive and integrative approach. Unlike the MDGs, which apply primarily to developing countries, the SDGs are universally relevant, applying to all countries regardless of their level of development. This universality underscores the interconnected nature of the modern world, where challenges and solutions are not confined within national boundaries [41]. The 17 SDGs encompass objectives such as eradicating poverty (Goal 1), ending hunger (Goal 2), ensuring healthy lives and promoting well-being for all ages (Goal 3), providing inclusive and equitable quality education (Goal 4), and achieving gender equality (Goal 5). Other goals focus on clean water and sanitation (Goal 6), affordable and clean energy (Goal 7), decent work and economic growth (Goal 8), industry, innovation, and infrastructure (Goal 9), reducing inequalities (Goal 10), sustainable cities and communities (Goal 11), responsible consumption and production (Goal 12), climate action (Goal 13), life below water (Goal 14), life on land (Goal 15), peace and justice (Goal 16), and partnerships for the goals (Goal 17) [16].

Each goal includes specific targets and indicators designed to measure progress over the 15 years from 2015 to 2030. These targets are comprehensive, detail-oriented, and aimed at catalyzing tangible outcomes. The SDGs emphasize the importance of collaborative efforts among governments, international organizations, civil society, and the private sector to tackle these complex issues effectively. Ultimately, the SDGs represent a blueprint for achieving a more viable and just world where all individuals can lead fulfilling lives in harmony with nature. Integrating these goals into national and international policies and practices is essential for realizing a sustainable future [42]. Embedding CSE within the SDGs framework acknowledges that sexual and reproductive health education is crucial for sustainable development. This approach recognizes that educating young people about their sexual and reproductive health is not just a matter of personal well-being but also a cornerstone for broader societal progress [21,27]. By integrating CSE with the SDGs, we can create a holistic framework that nurtures healthy, informed, and equitable communities, leading to sustainable development and the well-being of future generations.

SDG 1: no poverty and SDG 8: decent work and economic growth: Integrating CSE with the SDGs will address the interlinked challenges of poverty, education, and economic participation. Educated adolescents, particularly girls, are more likely to delay marriage and childbirth, stay in school longer, and pursue higher education or vocational training [43-45]. This delay in starting a family can significantly boost their economic prospects and contribute to breaking the cycle of poverty. Consequently, these individuals are better positioned to contribute positively to their communities and the global economy, which aligns with the objectives of SDG 1: no poverty and SDG 8: decent work and economic growth [46-48]. Beyond individual health and gender equality, aligning CSE with the SDGs contributes to broader societal goals. It promotes educational attainment by reducing drop-out rates related to early pregnancies and STIs. It also fosters economic growth by empowering individuals to make informed decisions about their health and lives, thus enhancing their potential for economic participation and productivity [47,49].

SDG 3: good health and well-being: Contributing to SDG 3, CSE plays a direct role in promoting health and well-being. CSE helps build a foundation for healthy and empowered individuals, equipping them with the necessary knowledge and skills to make informed decisions about their bodies and relationships. By providing accurate information about sexual health, this education helps prevent and control STIs and HIV/AIDS, reduce adolescent pregnancies, and promote safe practices [50,51]. Ensuring young people have access to this vital information supports their physical and mental health, contributing to healthier communities and reducing health disparities. Educated individuals are more likely to seek necessary health services, engage in safe sexual practices, and advocate for their rights, leading to healthier communities overall. Subsequently, it helps reduce the public health burden and healthcare costs [52-54].

SDG 4: quality education: CSE significantly intersects with SDG 4 on quality education. By promoting access to accurate and age-appropriate information on sexual and reproductive health within educational settings, CSE contributes to achieving SDG 4's targets related to inclusive and equitable quality education for all (UNESCO, 2018). CSE equips young people with the knowledge, attitudes, and skills to make informed decisions about their sexual health, relationships, and well-being [55]. Integrating CSE into the broader framework of quality education helps create an inclusive and supportive learning environment that addresses the holistic needs of students [56,57]. Moreover, CSE fosters an understanding of human rights, gender equality, and the importance of healthy relationships, aligning with SDG 4's aims to promote inclusive and lifelong learning opportunities for all [58]. Through its role in enhancing educational quality and inclusivity, CSE contributes to realizing the objectives of SDG 4 on a global scale.

SDG 5: gender equality: The intersection of CSE with SDG-5, which focuses on achieving gender equality and empowering all women and girls, is vital for promoting inclusive and equitable societies. CSE is crucial in advancing SDG 5 by challenging gender norms, addressing gender-based violence, promoting reproductive rights, and fostering gender-equitable attitudes and behaviors [55,59]. CSE empowers both girls and boys with the knowledge and skills to challenge traditional gender roles and stereotypes, promoting respect, equality, and consent within relationships [60]. By providing information on sexual and reproductive health, CSE helps to reduce gender-based health disparities, support reproductive rights, and advance women's and girls' overall well-being [61]. Furthermore, CSE offers a platform to address critical issues such as child marriage, female genital mutilation, and gender-based violence, which are significant obstacles to achieving gender equality [62,63]. Promoting CSE can contribute to an environment where women and girls can make informed decisions about their bodies, sexuality, and relationships, thus supporting the broader agenda of gender equality as outlined in SDG 5. Through its foundational support of gender-equitable values and behaviors, CSE aligns with SDG 5's aim and fosters an environment conducive to advancing gender equality and empowering women and girls globally [52,61-64]

SDG 13: climate action: The intersection of CSE with SDG 13 on climate action may not be immediately evident. Still, it is increasingly recognized as crucial for holistic sustainable development. CSE can significantly address SDG 13 by fostering a more climate-resilient future through population education, sustainable reproductive choices, and awareness of the interconnections between environmental sustainability and sexual health [16]. By integrating mise en scène sustainability into sexual education, CSE can promote responsible reproductive health choices, including family planning and understanding ecological impacts on population growth. Furthermore, CSE can contribute to more environmentally conscious behaviors by encouraging discussions around sustainable lifestyles, consumption patterns, and gender-responsive approaches to climate action [65]. Recognizing the intricate linkages between population dynamics, sexual health, and environmental sustainability, CSE encourages informed decision-making and an understanding of the ecological impact of these decisions. By framing sexual education within the context of environmental sustainability, CSE supports SDG 13 by fostering a population educated on the interconnectedness of sexual and reproductive health with environmental well-being [66]. In doing so, CSE aligns with the broader agenda of climate action and environmental sustainability as outlined in SDG 13.

SDG 17: creating partnerships for sustainable development: The intersection of CSE with SDG-17 emphasizes the critical role of partnerships and cooperation in achieving sustainable development. CSE is pivotal in advancing SDG 17 by fostering collaborative efforts among diverse stakeholders to promote inclusive, evidence-based sexual education programs [67,68]. Partnerships and cooperation are essential for the effective implementation of CSE initiatives. These programs often involve coordination among governments, educational institutions, civil society organizations, and the private sector [69]. By bringing together these stakeholders, CSE promotes collective action to provide young people with accurate and age-appropriate information about sexual and reproductive health, fostering a supportive and inclusive learning environment [70].

**Figure 4:**
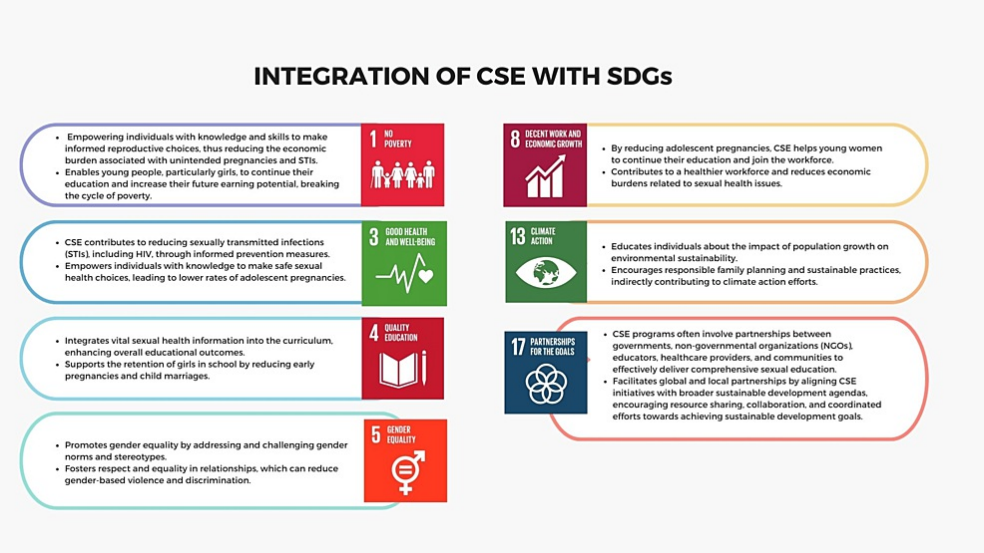
The integration of CSE with SDGs. CSE, comprehensive sexual education; SDGs, sustainable development goals Image credit: Nor Faiza Mohammed Tohit

Moreover, CSE offers a platform to address cross-cutting issues, including gender equality, human rights, and health, requiring collaboration across sectors and borders. Additionally, international cooperation can facilitate the sharing of best practices, resource mobilization, and capacity-building efforts to enhance the quality and reach of CSE programs globally [71,72]. Through its emphasis on collaboration and partnership, CSE aligns with SDG 17's intention and fosters an environment conducive to advancing sustainable development through cooperative action. By aligning sexual education with the SDGs, there is a potential to create synergies and partnerships across sectors, including education, health, and development, to ensure that young people receive quality, age-appropriate, and evidence-based sexual education that not only fulfills their rights but also contributes to broader societal well-being and sustainable development [73,74]. The integration of CSE with SDGs is shown in Figure 4.

Limitations of the review

This scoping review has several limitations that should be acknowledged. First, the search was limited to articles published in English, which may have excluded relevant studies in other languages, potentially skewing the geographic and cultural diversity of the findings. Second, the review only included literature published within the last 20 years, which could have omitted foundational studies that may still hold significant value in understanding long-term trends and impacts of CSE on sustainable development. Additionally, the reliance on electronic databases such as PubMed, Google Scholar, JSTOR, and Scopus may have excluded valuable grey literature or studies not indexed in these databases.

The selection process, although systematic, was subject to human bias, especially during the initial title and abstract screening stages. Furthermore, excluding non-peer-reviewed articles, conference posters, and editorials could have omitted emerging insights and innovative approaches not yet subject to peer review. Finally, the broad scope of sustainable development encompasses diverse and interconnected dimensions, making it challenging to address all relevant aspects within a single review comprehensively. Future research should consider expanding the inclusion criteria, incorporating non-English studies, and exploring diverse sources to provide a more holistic view of the interplay between CSE and sustainable development.

Future Research Opportunities in CSE and Sustainable Development Integration

**Figure 5:**
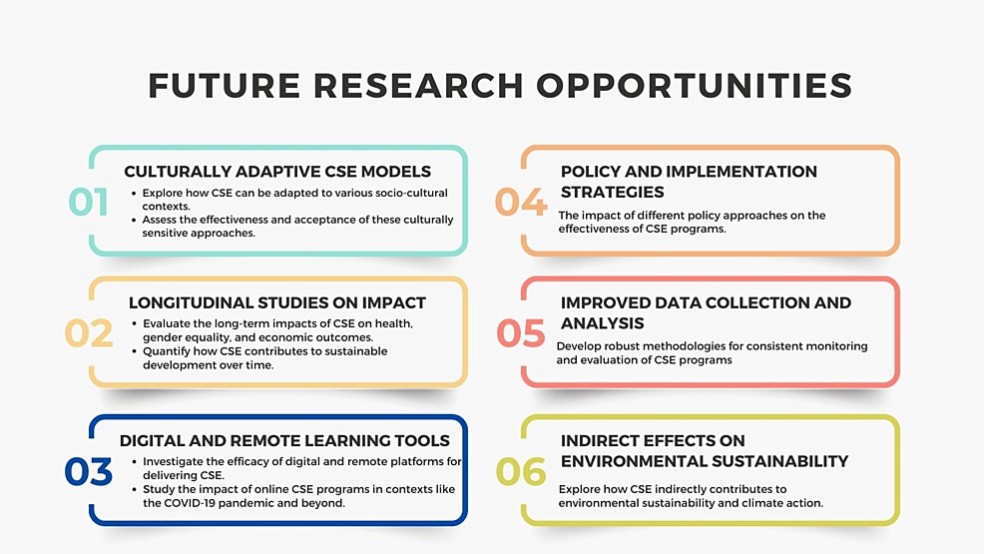
Future research opportunities. Image credit: Nor Faiza Mohammed Tohit

Future research opportunities for this review on CSE and its alignment with sustainable development are abundant and diverse, as shown in Figure 5. One critical area is the exploration of culturally adaptive CSE models that can be effectively implemented in various socio-cultural contexts, particularly in regions with strong cultural and religious norms [75,76]. Investigating how these adaptations affect the effectiveness and acceptance of CSE programs can provide valuable insights for policymakers and educators.

Additionally, longitudinal studies are needed to assess the long-term impacts of CSE on health outcomes, gender equality, and economic status. Such studies can help quantify the benefits of CSE in fostering sustainable development and empower stakeholders to make data-driven decisions. There is also a pressing need for research that evaluates the role of digital and remote learning tools in delivering CSE, especially during the COVID-19 pandemic and beyond [77-82]. Another promising area is the examination of multi-stakeholder partnerships and their efficacy in enhancing the reach and quality of CSE programs, including assessing the collaboration between governments, NGOs, and educational institutions. Furthermore, research can delve into the indirect effects of CSE on environmental sustainability, exploring how educated populations make informed decisions that contribute to climate action [65,66]. Finally, more comprehensive data collection and analysis methodologies should be developed to monitor and evaluate CSE programs' outcomes consistently across different regions, helping to identify best practices and areas for improvement [69].

**Figure 6:**
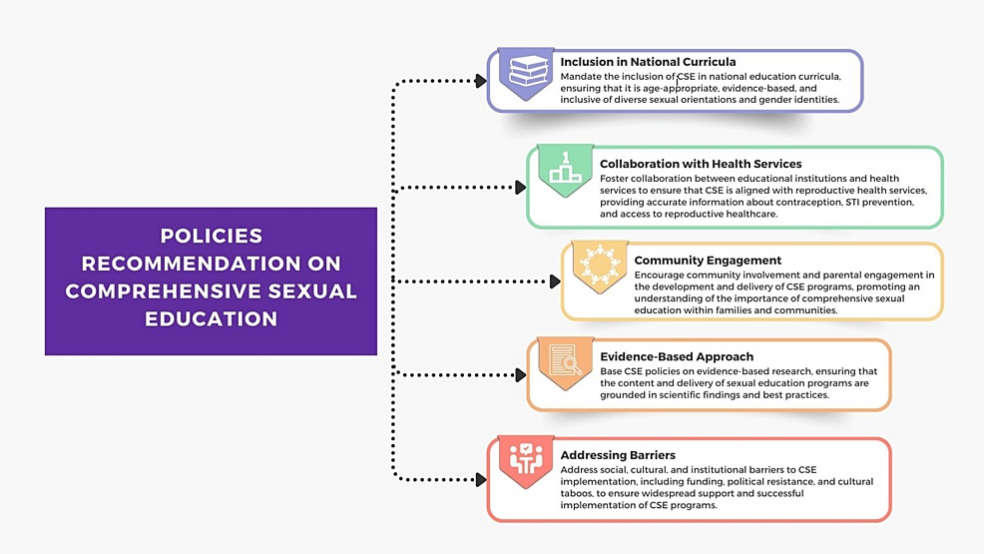
Policies recommendation on CSE. CSE, comprehensive sexual education Image credit: Nor Faiza Mohammed Tohit

Additionally, research focused on policy and implementation strategies could provide evidence-based recommendations for more effective integration of CSE in education systems globally [5,8,9]. These policy recommendations (Figure 6) are aimed at promoting the adoption and implementation of CSE, with a focus on promoting inclusivity, evidence-based information, and collaboration among stakeholders to ensure the well-being and empowerment of young people. Interdisciplinary research that bridges technology, education, public health, sociology, and environmental science is also encouraged to understand the holistic impacts of CSE, fostering interdisciplinary collaborations to enrich the understanding of CSE's role in sustainable development [82-85].

## Conclusions

The alignment of CSE with the SDGs is not merely a crossroads of two critical agendas but a profound integration that can drive significant progress toward healthier, more equitable, and sustainable futures. By recognizing and harnessing the synergy between CSE and the SDGs, we can empower young individuals, foster inclusive communities, and contribute to a global legacy of well-being and gender equality. This manuscript underscores the urgency and potential of this alignment, advocating for a concerted effort to harmonize educational and developmental objectives in pursuit of a better world for all.
